# Mode of Managing Ocular Inflammations

**Published:** 1802-03

**Authors:** D. Whyte

**Affiliations:** Bay of Aboukir


### *Mode of managing Ocular Inflammations*; By Dr. WHYTE.

THAT expofure of the naked eye to the intenfe heat and vivid rays of a nearly vertical fun, fhould in fome cafes occafion an expanfion of the humours and dilatation of the veffels of that delicate organ, is in the nature of things not to be doubted.

Where the conftitution is naturally delicate, where it has been formed in another climate, and is not yet fufficiently affimilated to the new one, fuch expanfion and dilatation will, on the requifite expofure, more certainly enfue.

To guard againft fuch expofure conftitutes therefore a material part of ocular prophylaxis. It is to be effected by fhading the eyes from the influence of the vertical ray, by means of broad brimmed hats, long vizored helmets, or what would anfwer beft of all, the tamata of Taheite, a garland compofed of palm leaves with a plated projecting front piece; this of all coiffures is the moft eminently fuitable, becaufe it does not debilitate the head by exceffive heat, as do the ponderous helmets of European foldiers, or the equally ponderous turbans of pious Muffelmen.

Neither the cocked hat of modern Europe, nor the mouchoir fo generally worn by the lower claffes of the French and Spaniards, as well at home as in their American poffeffions; nor the turban of the fwarthy inhabitants of Africa or Afia, afford any fhade or protection to the eyes.

Accordingly, all thofe enumerated claffes, in every variety of climate and foil, are exceedingly liable to ocular inflammations, and to their remote confequences, fpecks and cataracts.

In fome of thofe countries too, the influence of the folar ray is much increafed by inhabiting a houfe having white-wafhed walls, or where the eye of luxury is dazzled and deftroyed by the luftre of elegant but fuperfluous chandeliers, or by the reflexion from a multiplicity of fplendid mirrors. Where fuch caufes have alone operated, and application has been made for medical affiftance in the firft ftage of the difeafe, it is for the phyfician or furgeon to determine whether the difeafe he is about to treat confifts in a mere dilatation of the humours, or in an inflammation of the tunics, or whether it is a mixed cafe, and both circumftances are combined.

In the firft fpecies there is frequently no perceptible inflammation; but, by an enlargement of the aqueous humour, and the anterior fection of the orbit, the focus of concentration falls behind the retina. An indiftinct image is formed upon it even in day-light, while towards evening the lucid rays proving fcanty and feeble, feem blended and confufed, and vifion is completely interrupted.

I am acquainted at prefent with a cafe of this kind, which was induced by the patient indulging himfelf with an afternoon's nap, in the open air, on the rock of Gibraltar, laying on his back, and with his eye-lids confiderably open.

That this imprudent expofure did not at the fame time occafion an inflammation of the blood veffels that ramify and run along the furface of the tunica albuginea, depended on the abfolute tone and ftrength of thofe veffels, and on their being relatively ftronger, and lefs expanfible, than the fubjacent tunics or contained humors.

This man recovered without having had recourfe to medical affiftance.

By avoiding for fome time the ftimulus of light, or, more correctly fpeaking, the expanfive power of heat, the humors infenfibly collapfed, and the tunics refumed their original and healthy tone.

Had this perfon, however, been a patient of mine, I would have affifted the flow progrefs of Nature, by keeping the ball of the eye conftantly moiftened with a cloth or rag dipped in cold water, or fome gently aftringent collyrium, as aqua litharg. acetati, aqua zinci, vitriolati, &c.

I would have alfo touched the ball of the eye, morning and evening, or oftener, with fome aftringent and ftimulating tincture, as that of cinchona, or of opium; or, to fuperfede every other remedy, and ftrike at once to the root of the difeafe, I would have pierced through the tunics with a couching needle, and entering the pofterior chamber of the aqueous humor by an incifion parallel to and behind the iris, permitted an outlet proportioned to the exifting expanfion.

No danger need be apprehended from this operation; it is even in this manner I am accuftomed to extract the cataract. It is a mode that poffeffes many advantages. I have performed it frequently, ever with impunity, and often with fuccefs.

When, from the operation of light and heat alone, the veffels of the tunica albuginea have alone fuffered, a fteady and conftant employment of collyria, in the extenfive manner recommended, with the diurnal or more frequent application of fome ftimulating tincture, will in moft cafes, where the eye labours under no conftitutional difeafe, and we have been confulted at an early period, effect in very few days a complete cure, while avoiding of the exciting caufe will fecure againft relapfe.

The momentary pain arifing from the application of the ftimulating tincture is often exceedingly acute, yet fo fenfible are thofe thofe who have once experienced it, of its great utility, that the moment I enter fome fhips where, from incorrigible negligence in not wearing hats or bonnets, this difeafe is exceedingly frequent, I am furrounded by a circle of foldiers' wives and children entreating to be touched.

I may likewife add, that when leeches can be procured we fhould do well to apply them in fome cafes, repeating them as circumftances may require, and applying them as near as poffible to the feat of the difeafe.

In fuch cafes, when leeches cannot be obtained, we may open the jugular vein, or, ftill better, the temporal artery.

In fevere cafes I have fometimes fcarified inflamed eyes, but after fcarification ecchymofis often accedes, and the eye may remain blood-fhot, a difagreeable circumftance, for weeks together.

Befides, the application of ftimulating tincture and aftringent collyria has proved, in my practice, fo invariably beneficial as to fuperfede, in my opinion, every other mode of treatment.

If I hefitate to deform the eye by fcarifications, the moft indifpenfible neceffity could alone determine me to disfigure the face by difagreeable and difgufting veficatories.

Sometimes, when from the extreme fenfibility of the vifual organ, and its vicinity to the primum mobile, the primum mobile itfelf is affected, I am taught by the common rules of prudence to obviate danger, and afford relief by an immediate diminution of the circulating mafs, moderate or copious, and repeated or not, according to the urgency of exifting circumftances.

It is almoft unneceffary to add, that the ftate of the bowels muft be likewife attended to.

So much for thofe inflammations of the eyes that originate from improper expofure to heat and light alone.

The inflammations fo frequent in Syria and Egypt have often another and more pernicious fource.

By means of proper coiffures we can protect the moft tender of organs from the meridian rays of a fcorching fun; but what human invention, what undifcovered amulet, can fecure the eye of him who inhabits a wildernefs of fand, from the intrufion of its minute and infinuating particles, when that wildernefs is, like the waters of the ocean, agitated by the winds?

Of one thing however we may reft affured, that till the irritating particles are fairly expelled, totally diffolved, or completely encyfted, no mode of treatment can operate a cure.

Nature, ftudious for the fafety of all her productions, has been eminently provident of this the moft exquifite and beautiful of all her works. She has formed the eye-lids to fecure the the included orbs againſt the impulſe of foreign bodies; and the tears, ſecreted for the purpoſe of moiſtening the eye, and maintaining it in a condition ſuitable to the performance of its peculiar functions, are on the ſmalleſt irritation from a foreign body, ſecreted in increaſed quantity, and aſſiſt in the ſolution or expulſion of ſo dangerous an inmate.

It is the buſineſs of the philoſophical practitioner to ſecond the efforts of Nature, and endeavour to improve upon her curative indications.

In ſome countries it is a common practice among the peaſants to attempt the removal of motes in the eye by wiping the ball gently with a feather, or employing a damſel to ſkim it with her tongue; but both modes are rude, and frequently ineffectual. It is more proper, in my opinion, to tread in the footſteps of Nature, and endeavour to cleanſe the eye by copious ablution, by a forcible and well directed ſtream, which pervading every receſs, ſhall in its progreſs ſweep every foreign particle before it. Accordingly, in ocular inflammations ariſing from this ſource, or where ſuch a ſource is at all ſuſpected, I enjoin the eye to be inſtantly ſyringed.

Not unfrequently the patient is ſenſible of the expulſion of the irritating particle during the operation. Sometimes, however, it only ſhifts its ſeat, and one or more repetitions are required. In every other reſpect the inflammation is to be treated as if proceeding from the influence of heat and light alone.

Should any gentleman do me the honour to make trial of the methods I have here recommended, I flatter myſelf he will find his account in it, and neither have occaſion to accuſe me of preſumption, nor himſelf of temerity.

---

### *Postscript to Dr.* WHYTE'*s Paper on Ocular Inflammations.*

SINCE writing the preceding account of my mode of treating ocular diſeaſes, I begin to think better of ſcarification, and to practiſe it oftener.

I have even met with ſeveral caſes where, I apprehend, ſuffuſion would have been the conſequence of its omiſſion.

I have alſo ſeen, ſince writing the original paper, ſome Eſſays on Ophthalmia in the Egyptian Decade, with which I have been favoured by Sir Sidney Smith, and am much ſurpriſed that in the enumeration of remedies by one of their principal phyſicians, ſcarification is not ſo much as mentioned.

It is obſerved indeed by Citizen Bruant, in a different paper, that the natives employ, with much advantage, topical blood-letting at the external canthus, by which, I ſuppoſe, he means ſcarification; but, he adds, that the French could not, or or had not yet adopted it. Citizen Bruant fuppofes that fome *ophthalmies* proceed from bilious accumulations in the primæ viæ.

If the eye is fufficiently predifpofed, or the fever fufficiently violent, this may no doubt happen.

This fpecies, as he chufes to term it, he treated moft fuccefsfully by emetics and purgatives.

General phlebotomy, he fays, was contra-indicated in this fecond fpecies by the prefence of bile; and in the firft, or local fpecies, arifing from duft, &c. by the debility induced upon the troops by the hardfhips of a nine year's war.

Like preceding medical conjurers, he furprifes us with ftill a third fpecies, arifing, he fays, from nervous irritability.

The number of different and difcordant fubftances that have been ufefully employed as collyria, demonftrates to me that they all act upon one common principle, viz. the principle of ftimulation. Still I cannot place perfect confidence in Dr. Savarefi's obfervations, when he informs us, in one page, that powdered fulphat of alumine (common alum), invariably produced ophthalmia; and, in a fubfequent one, that by blowing a powder, compofed of it, fugar candy, and nitrated kali (nitre), upon the ball of the eye, he has invariably fucceeded in the removal of incipient fpecks.

Savarefi informs us, that of one thoufand patients whom he treated, two only became totally blind, and two loft an eye.

This was great fuccefs; and much certainly depended on an early application.

He frequently applied blifters to the nape of the neck, and always ufed ftrong ftimulating collyria.

His cures were, however, exceedingly protracted, being in general from three weeks to two months; and might, I apprehend, have been much fhortened had he occafionally fcarified, as I now do; and had he touched the ball of the eye, once or twice a day, with a hair pencil dipped in fome ftimulating tincture.

Although the French have had fo excellent an opportunity, they do not appear to have acquired a very accurate knowledge of the nature and caufes of ocular inflammation. In attributing the difeafe to this or that faline powder, they ought to have reflected that any powder finding admiffion into the eye, will there produce irritation and pain, and, finally, more or lefs inflammation, till fuch time as its particles become encyfted, or until they are diffolved or neutralized, or expelled by the lachrymal fecretion.

If nitre is found to be lefs irritating than alum, we may prefume it to be more foluble. And if any powder is more prejudicial judicial than another, I believe it to be lime. The Egyptian mafons have not the art to avoid its influence, and the French phyficians report that moft of them are blind. But why contend concerning faline powders, when a fingle grain of fand, of which there would not appear to be any fcarcity in the lower parts of the Egyptian atmofphere, will fufficiently explain the phenomenon; and when, in a thoufand other cafes, unguarded expofure of the eye to intenfe folar heat will not only furnifh fufficient explanation, but the difeafe may be often traced to fuch a fource.

Dr. Savarefi talks of fthenic and afthenic ophthalmia; but I difagree with him and many others on this fubject; fo much fo, that I do not even believe there is in exiftence fuch a thing as fthenic topical inflammation. There may exift a general increafe of vafcular action; but I contend that no accumulation can take place in any individual part or organ, unlefs fuch part or organ has, conftitutionally or incidentally, lefs ftrength, or tone, or refiftance, abfolute or relative, than the other parts where no fuch accumulation has acceded. If the local predifpofing debility, inherent or induced, is fufficiently great, ophthalmia may be excited without any increafe of general vafcular action. An increafe of general vafcular action may fometimes be neceffary to excite topical inflammation; but the veffels of the part muft at the time poffefs lefs relative tone, otherwife they would not yield to the diftending power. Such local and predifpofing debility is produced by no means fooner or fo much as by heat. Hence expofure of the eye to the direct influence of the folar rays, will ever predifpofe to ophthalmia; and, where the predifpofition is fufficiently great, the difeafe will be excited without any oftenfible caufe, and without any increafe of vafcular action.

There may be more or lefs decreafe of tone in the inflamed part; a decreafe that is recoverable, and one that is not.

That fthenic topical inflammation, properly fpeaking, does not actually exift, would appear, not only from theoretic reafoning, but is confirmed by the prefent improved practice of the Englifh furgeon, who applies tonics and aftringents to every external part that is inflamed. Could the phyfician act fimilarly with internal inflammations, we might entertain fome hope of the univerfal panacea being at laft difcovered.

The reafoning I have employed is confonant to the eftablifhed laws of hydroftatics, and to thofe of the animal œconomy; confequently, topical tonics are indicated in all cafes of ophthalmia; and in many, general debilitating powers.

I agree with the French phyfician, that expofure to nocturnal dews may ferve as an exciting caufe; and I apprehend I

furnifh furnifh a more definite idea of the phenomenon when I ftate, that, by expofure to nocturnal cold, the fluids, receding from the major part of the fuperficies, are forced to concentrate themfelves in that part or organ, internal or external, laboring under the greateft abfolute or relative debility; — in this cafe, the eye, which it is therefore of much confequence to fhade from the fun. But, in fhading the eye, I do not conceive that there is an equal neceffity to have the head covered. It is recorded by an ancient hiftorian, (Herodotus, I believe) that on the fields where battles had been fought, the head of an Egyptian could be diftinguifhed from that of a Parthian. The latter, which had been habitually invefted with the many-folded tiara, was thin, light, and femi-tranfparent. The former had gone bare headed when alive; and his fkull, hardened and baked in the fun, was thick, opake, and ponderous. It is, perhaps, on a fimilar principle that we ought to account for the thick fkins of the inhabitants of hot climates, particularly Negroes.

Convinced of the impropriety of retaining the head in a perpetual ftate of perfpiration, a ftate that renders the fmalleft expofure, by uncovering, extremely hazardous, I have lately, although expofed the greater part of the day in an unfhaded boat, left off wearing a hat, and fubftituted a fmall *tamatà* of green filk to intercept the fun from my eyes. It is, in my eftimation, a confiderable luxury to preferve the head free of perfpiration; and from the want of a hat, I feel no inconvenience whatever. I have ftill another, and perhaps more important purpofe in view by leaving off a hat; which is, to demonftrate to the world, that the difeafe fantaftically termed Coup de Soleil, proceeds from very different caufes than expofure of the head to folar heat; — but of this in another place, and at another time.

Citizen Bruant obferved in the courfe of his Egyptian practice, that Ophthalmia and Dyfentery often alternated with one another; and for their removal, he fometimes applied blifters to the calves of the legs. The fluids, fublimed by heat, and fometimes repreffed from the furface by cold, evidently fought for an outlet. To blifters, therefore, I would have preferred fetons, or iffues, or occafional phlebotomy. In inveterate Ophthalmia, nothing can equal the efficacy of a feton or iffue at the nape of the neck; yet no *Franko-Egyptian* phyfician I have yet feen, fays a word either of fetons or iffues.

---

### Forms of Collyria.

No. 1. Of corrofive fublimate, 6 grains; ardent fpirit of any any kind and pure water, of each 6 oz. Mix, and keep the eye conftantly moiftened with a cloth dipped in it.

No. 2. Of laudanum, a tea fpoonful; ardent fpirit of any kind, vinegar, water, of each 4 oz. Mix, &c.

To thefe two forms, particularly No. 2, I am inclined to give a decided preference.

No. 3. Of alum, 1 drachm; water, 8 oz.

No. 4, 5, and 6. Collyria fimilar to No. 3, may be made with an equal proportion of nitrated kali, (common nitre); of vitriolated zinc, (white vitriol); or of ceruffa acetata, (fugar of lead.)

No. 7. Goulard's extract, a tea fpoon full; vinegar, water, of each 4 oz. Mix, &c.

No. 8. Vinegar, pure water, equal parts. Mix, &c.

No. 9 and 10. When none of the preceding articles can be procured, falt, or even frefh cold water, may ferve as an imperfect fubftitute.

D. WHYTE, M.D.

*Bay of Aboukir, July* 8, 1801.

